# Neural correlates of the age-related changes in motor sequence learning and motor adaptation in older adults

**DOI:** 10.3389/fnhum.2013.00142

**Published:** 2013-04-17

**Authors:** Bradley R. King, Stuart M. Fogel, Geneviève Albouy, Julien Doyon

**Affiliations:** Functional Neuroimaging Unit, Centre de Recherche de l'Institut Universitaire de Gériatrie de Montréal, University of MontrealMontreal, QC, Canada

**Keywords:** aging, motor learning, consolidation, adaptation, procedural memory, neuroimaging, striatum, cerebellum

## Abstract

As the world's population ages, a deeper understanding of the relationship between aging and motor learning will become increasingly relevant in basic research and applied settings. In this context, this review aims to address the effects of age on motor sequence learning (MSL) and motor adaptation (MA) with respect to behavioral, neurological, and neuroimaging findings. Previous behavioral research investigating the influence of aging on motor learning has consistently reported the following results. First, the initial acquisition of motor sequences is not altered, except under conditions of increased task complexity. Second, older adults demonstrate deficits in motor sequence memory consolidation. And, third, although older adults demonstrate deficits during the exposure phase of MA paradigms, the aftereffects following removal of the sensorimotor perturbation are similar to young adults, suggesting that the adaptive ability of older adults is relatively intact. This paper will review the potential neural underpinnings of these behavioral results, with a particular emphasis on the influence of age-related dysfunctions in the cortico-striatal system on motor learning.

## Introduction

The learning of new motor skills, as well as the modification of previously learned skills, is necessary for both the performance of everyday activities and the implementation of neurorehabilitative training programs following brain injury (i.e., stroke). As the average age of the world's population continues to rise, an increased comprehension of the relationship between aging and motor learning will be fundamental to both our understanding of how the motor system functions and how to treat motor deficits. Accordingly, the overarching purpose of this paper is to provide a review of the extant literature investigating motor learning, as well as the associated neural underpinnings, in older adults. To achieve this aim, we will examine the results from research investigating the behavioral and neural correlates of the two most frequently studied motor learning paradigms: motor sequence learning (MSL) and motor adaptation (MA).

MSL involves integrating the temporal structuring of a series of actions into a coherent unit, whereas MA requires the modification of previously learned movements in response to changes in the organism, task or environment. Both MSL and MA have been extensively studied in young subjects and are thought to follow several distinct phases: (1) a fast initial, within-session learning phase where the magnitude of the behavioral improvements is substantial; (2) a slow, across-session phase in which smaller behavioral improvements are evident over days, weeks, or months of practice; and, (3) an intermediate phase that occurs between practice sessions in which the motor memory is transformed from an initial labile trace to a more stable and resistant form (e.g., Karni et al., 1995, 1998; Doyon et al., 2003; Krakauer et al., 2005). Although the behavioral and neural correlates of MSL and MA are relatively similar during early learning, there is ample evidence indicating that they differ when performance becomes asymptotic and motor memory consolidation begins (for reviews, see Doyon et al., 2003, 2009a; Doyon and Benali, 2005). Indeed, the initial fast learning phase of both MSL and MA elicits widespread activation in cortical and subcortical structures, including the basal ganglia, cerebellum, the supplementary motor area (SMA) as well as the primary motor (M1), premotor (PM), and prefrontal (PFC) cortices. However, consolidation and retention of learned motor sequences is thought to be dependent on the cortico-striatal network, whereas consolidation and retention following MA is predominantly considered a function of the cortico-cerebellar system (Krebs et al., 1998; Penhune and Doyon, 2002; Ungerleider et al., 2002; Doyon et al., 2003, 2009a; Doyon and Benali, 2005; Galea et al., 2010; Landi et al., 2011).

Behavioral studies examining the influence of aging on MSL and MA have consistently reported the following pattern of results: (1) the initial, fast learning phase of MSL appears to be relatively spared by the aging process except under conditions of increased task complexity (e.g., Curran, 1997; Feeney et al., 2002; Howard et al., 2004; Bennett et al., 2007; Rieckmann and Bäckman, 2009); (2) older adults demonstrate impairments in the consolidation of learned motor sequences (e.g., Spencer et al., 2007; Brown et al., 2009; Nemeth and Janacsek, 2010; Nemeth et al., 2010; Fogel et al., 2012; Wilson et al., 2012); and, (3) older adults demonstrate deficits during the exposure phase of MA paradigms; however, the magnitude of the aftereffects in the post-exposure phase is comparable to that of young adults (e.g., McNay and Willingham, 1998; Fernandez-Ruiz et al., 2000; Bock, 2005; Bock and Girgenrath, 2006; Seidler, 2006, 2007a; Heuer and Hegele, 2008; Hegele and Heuer, 2010; Anguera et al., 2011). Although seemingly distinct, these behavioral results may be manifestations of common age-related degradations in the structure and functioning of relevant neural substrates and networks. This paper will discuss the influence of the aging brain on the impairments highlighted above, with a particular emphasis on the cortico-striatal networks critical for the different phases of MSL and MA.

This review is organized into four sections. Following this introductory section, we provide a brief overview of MSL and MA, emphasizing behavioral results and neural correlates from research in young adults. The third section highlights motor learning in older adults^1^, and discusses evidence linking the behavioral deficits to age-related changes in relevant neural substrates; specifically the cortico-striatal network. The fourth section will then provide general conclusions.

## Motor learning in young adults: an overview

### Motor sequence learning (MSL)

#### Behavioral results

MSL refers to the process by which simple, stereotyped movement elements come to be performed effortlessly as a unitary well-rehearsed sequence. This type of procedural learning has been investigated with a variety of different laboratory-based protocols; the most common requires participants to use the fingers of the right or left hand to either press buttons on a keyboard, or to lightly touch one's own thumb in a precise and sequential order. The sequence of movements may be explicitly (e.g., Karni et al., 1995; Korman et al., 2003) or implicitly learned (e.g., Robertson et al., 2004b), self-initiated (e.g., Karni et al., 1995), cued by visual or acoustic stimuli (e.g., Nissen and Bullemer, 1987), or interleaved with random movements (e.g., Howard and Howard, 1997). Despite these methodological differences, participants typically increase the velocity of their finger movements and decrease the interval between successive key presses with practice, resulting in a decrease in the duration to complete the repeated sequence (a measure of speed) and the number of errors made (a measure of accuracy). These behavioral improvements are indicative of learning the sequence and can also be used as indices of memory consolidation when performance is subsequently retested.

Although a detailed characterization of the initial acquisition of movement sequences is critical to our understanding of motor learning, it is equally important to understand how the retention of these newly acquired memories occurs over longer periods of time. In the context of implementing interventions designed to ameliorate age-related declines in motor performance or to increase functional mobility following neurological injury, improvements in motor functioning must be maintained beyond the conclusion of the training session. Experimental protocols typically assess retention by having participants return to the laboratory after a period of no practice to perform the same motor sequence. Retention is then quantified by making various comparisons across the different experimental sessions. In the interest of clarity, this review will adopt the following terminology that is used in the extant literature to characterize retention. The term “savings,” although more commonly used in the MA literature, refers to significantly better performance (i.e., reduced errors or faster rate of learning) during the early portion of the retention session as compared to the early portion of initial training (Krakauer, 2009). “Off-line gains” refers to better performance in the early portion of the retention session as compared to the end of the initial training session (e.g., Robertson et al., 2004a)^2^. And finally, the term “consolidation” refers to the process by which an initially labile memory trace becomes transformed into a more stable, enduring memory (McGaugh, 2000; Walker et al., 2003; Krakauer and Shadmehr, 2006). Consolidation may be reflected by off-line gains, maintenance of a trace across testing sessions as well as resistance to interference from competing memories (Robertson et al., 2004a; Walker, 2005). Critically, previous research in young adults has demonstrated substantial savings and off-line gains following periods of non-practice of a motor sequence for several hours up to 1 year (Karni et al., 1995, 1998; Penhune and Doyon, 2002; Walker et al., 2002; Romano et al., 2010).

The magnitude of the savings and off-line gains in young adults is enhanced by a period of sleep during the interval between initial training and retention. More specifically, both nighttime sleep and a daytime nap result in significant increases in off-line learning and resistance to interference from a competing memory trace as compared to an equivalent period of wakefulness (Walker et al., 2002, 2003; Walker and Stickgold, 2006; Korman et al., 2007; Nishida and Walker, 2007; Doyon et al., 2009b; Debas et al., 2010). There is also growing evidence to suggest that stage 2 sleep, and sleep spindles in particular, are involved in this consolidation process (Fogel et al., 2007; Nishida and Walker, 2007; Morin et al., 2008; Barakat et al., 2011, 2012). Sleep spindles are short synchronous bursts of neuroelectrical activity between 12 and 15 Hz that propagate through the thalamocortical loop (Steriade, 2006; Bonjean et al., 2011). Perhaps most importantly for the context of this review, sleep spindles are thought to be involved in long-term synaptic plasticity, providing an explanation for their role in the consolidation of learned motor sequences (for review, see Fogel and Smith, 2011).

Sleep-dependent consolidation has consistently been reported in explicit MSL paradigms where the sequence of elements to be performed is explicitly provided to the participants either prior to or throughout training (e.g., Korman et al., 2007; Debas et al., 2010; Albouy et al., 2013a). Conversely, implicit sequence learning paradigms typically employ some variant of the serial reaction time (SRT) task where participants press a button with the appropriate finger that corresponds to a specific visual stimulus presented on a computer screen. Unbeknownst to the participants, the sequence of stimuli (and thus corresponding finger movements) follows a repeating pattern or an underlying structure. The role of sleep in the consolidation of implicit motor sequence memories remains controversial as some studies have reported no influence of sleep (Robertson et al., 2004b; Song et al., 2007; Nemeth et al., 2010) whereas others have demonstrated sleep-dependent benefits (e.g., Albouy et al., 2008). The reasons for these inconsistent findings remain unknown, although some insights have been offered based on the recruitment of relevant neural substrates, a topic that is a focus of the subsequent section.

#### Neural correlates

The neural substrates underlying MSL in young adults have been extensively characterized (Grafton et al., 1995; Penhune and Doyon, 2002; Ungerleider et al., 2002; Doyon et al., 2003, 2009a; Doyon and Benali, 2005; Penhune and Steele, 2012) and are thus briefly summarized here. The initial acquisition phase of MSL elicits widespread activation, including, but not limited to, the basal ganglia, cerebellum, hippocampus as well as relevant cortical areas (e.g., SMA, M1, PFC, and PM cortex). However, the relative contributions of these different structures change as a function of learning. Activity in the striatum collectively increases while activity in the cerebellum decreases with practice, especially when behavioral performance is asymptotic (Grafton et al., 1995; Doyon et al., 2002; Penhune and Doyon, 2002). Within the fronto-striatal networks, it has been suggested that the caudate-DLPFC circuit as well as the rostrodorsal (associative) regions of the putamen are involved early in the learning process and are critical for acquiring an accurate sequence representation (Jueptner et al., 1997; Lehericy et al., 2005). By contrast, activity in the caudoventral (sensorimotor) areas of the putamen increases as a function of practice, suggesting that this region is involved in the execution of well-learned or automatic sequences (Jueptner et al., 1997; Lehericy et al., 2005). Independent of its role in motor execution, the cerebellum is especially critical for early sequence learning, not only for error detection and correction, but also in the acquisition of sequence knowledge (Seidler et al., 2002; Orban et al., 2010; Steele and Penhune, 2010). Last, the long-term storage of the motor memory is thought to be dependent on a distributed cortico-striatal network (Karni et al., 1995, 1998; Penhune and Doyon, 2002; Penhune and Steele, 2012).

The hippocampus has traditionally received very little attention in MSL and other procedural memory tasks as its function has been considered limited to declarative memory or tasks involving explicit learning mechanisms. More recently, however, the hippocampus has been implicated in both the initial learning and memory consolidation phases regardless of whether the sequences are implicitly or explicitly learned (Schendan et al., 2003; Albouy et al., 2008; Fernández-Seara et al., 2009; Gheysen et al., 2010). More particularly, activity in both the striatum and hippocampus during initial MSL (Albouy et al., 2008), as well as their functional interactions (Albouy et al., 2013b) have been described to predict subsequent consolidation processes. Rather than a distinction based on the implicit or explicit nature of the learning, recruitment of the hippocampus appears to depend on the *type* of information learned. Rose et al. (2011) demonstrated that bilateral hippocampal activation was evident only during learning of the perceptual, but not motor, component of a sequence. This result is analogous to recent research in our own laboratory suggesting that the hippocampus appears to be critical for the learning and consolidation of an allocentric, spatial representation of a sequence whereas the striatum is more involved in the learning and consolidation of an egocentric, motor representation (Albouy et al., 2012, 2013a).

Interestingly, consolidation of the allocentric, and presumably hippocampal-dependent, representation was enhanced by sleep whereas consolidation of the egocentric representation was not (Albouy et al., 2013a), suggesting that the recruitment of the hippocampus may be critical for sleep-dependent consolidation. This link between the hippocampus and sleep-dependent consolidation has also been used to explain the conflicting results investigating the role of sleep in *implicit* sequence learning (Section Behavioral results) (Song et al., 2007). Specifically, explicit, as compared to implicit, sequence learning is thought to rely more heavily on the hippocampus; thus, increasing the probability of sleep-dependent consolidation. It should be emphasized that this hypothesis certainly warrants further investigation because: (1) implicit sequence learning results in significant hippocampal activation (Schendan et al., 2003; Albouy et al., 2008; Gheysen et al., 2010); and, (2) sleep-dependent effects have been previously observed in implicit learning paradigms (Albouy et al., 2008).

Collectively, these results from neuroimaging research indicate that the hippocampus and both the cortico-cerebellar and cortico-striatal systems are involved in the initial learning of a movement sequence; however, consolidation and long-term retention are functions of the hippocampus and cortical-striatal network.

### Motor adaptation (MA)

#### Behavioral results

Movements need to be modified in response to changing conditions, such as when muscles are fatigued, when the dynamics of the end effector have changed as a result of growth or development or in response to bodily or brain injury. This adaptation process is typically examined by manipulating conditions in the environment in which participants move, specifically, by introducing visuomotor distortions (e.g., Kagerer et al., 1997) or mechanical perturbations (e.g., Shadmehr and Mussa-Ivaldi, 1994) during the execution of goal-directed movements. During initial exposure to a perturbation, participants typically make within-trial, feedback-dependent corrections (Thoroughman and Shadmehr, 1999). However, with continued exposure, these corrective responses are utilized in a feed-forward process, altering the initial motor commands of subsequent movements (Shadmehr and Mussa-Ivaldi, 1994; Thoroughman and Shadmehr, 1999). This feed-forward update becomes apparent when the perturbation is abruptly removed and subsequent movement paths are distorted in the direction opposite to that of the imposed perturbation (i.e., a clockwise visuomotor distortion would result in counter-clockwise movement trajectories). These distorted trajectories, in the absence of external perturbations, are referred to as aftereffects and provide a measure of the level of adaptation acquired during the exposure conditions (Shadmehr and Mussa-Ivaldi, 1994; Kagerer et al., 1997).

If young adults are re-exposed to the same perturbation after a time delay, the magnitude of the errors is decreased and the rate of adaptation is substantially increased, indicating savings in performance (Brashers-Krug et al., 1996; Shadmehr and Brashers-Krug, 1997; Shadmehr and Holcomb, 1997; Krakauer et al., 2005; Krakauer and Shadmehr, 2006; Krakauer, 2009). Yet in contrast to memory consolidation following MSL, the influence of sleep on consolidation following MA is less clear. Tononi and colleagues have demonstrated that sleep not only enhances MA consolidation in young adults, but the magnitude of the off-line improvements is correlated to the amount of slow wave activity (<4 Hz) in the right parietal region (Huber et al., 2004; Landsness et al., 2009). Conversely, research from our own group has demonstrated equivalent savings following periods of sleep and wake (Doyon et al., 2009b; Debas et al., 2010). These data are consistent with previous literature indicating that the passage of time, with or without sleep, is sufficient for MA savings (Brashers-Krug et al., 1996; Shadmehr and Brashers-Krug, 1997; Shadmehr and Holcomb, 1997, 1999; Krakauer et al., 2005). Last, sleep deprivation in young participants has been shown to have no detrimental influence on savings in performance but does deteriorate stabilization of the memory trace (Donchin et al., 2002; Albouy et al., 2013c). Although further research is certainly necessary, the majority of the evidence to date suggests that time in the wake state is necessary, but sufficient for MA consolidation to occur, and that sleep does not offer additional benefits for consolidation.

#### Neural correlates

Adapting or modifying movements in response to sensorimotor perturbations has largely been considered a function of the cerebellum. The cerebellum generates predictions of future states computed based on efferent copies of descending motor commands (Barto et al., 1999; Bastian, 2006; Miall et al., 2007; Nowak et al., 2007; Tseng et al., 2007; Miall and King, 2008). Discrepancies between actual and predicted states are then used as error signals that drive the adaptation process by altering the synaptic weights between the posterior parietal cortex (PPC), critical for specifying spatial information about both the end effector and desired target, and M1 (Tanaka et al., 2009). These error signals are ideal for supervised learning algorithms, a type of learning thought to be implemented in the cerebellum (Doya, 2000). Additional support for the role of the cerebellum in MA comes from both patient and imaging studies. Patients with cerebellar damage have demonstrated substantial deficits in sensorimotor adaptation (Martin et al., 1996; Smith and Shadmehr, 2005; Rabe et al., 2009; Criscimagna-Hemminger et al., 2010; Werner et al., 2010; Donchin et al., 2012) and studies using PET and fMRI have repeatedly shown extensive cerebellar activation during MA in healthy adults (Krebs et al., 1998; Imamizu et al., 2000; Nezafat et al., 2001; Seidler et al., 2006; Albouy et al., 2013c). Cerebellar activation can even predict the amount of subsequent savings in performance (Debas et al., 2010; Albouy et al., 2013c) and is also thought to be involved in delayed recall assessments, suggesting that the cerebellum is involved in the acquisition, consolidation and long-term retention of MA (Shadmehr and Holcomb, 1997; Imamizu et al., 2000; Nezafat et al., 2001; Della-Maggiore and McIntosh, 2005; Debas et al., 2010).

The basal ganglia also contribute to MA as research in Parkinson's disease (PD) has revealed that patients demonstrate substantial performance deficits, particularly when the magnitude of the movement errors is large as in abruptly introduced visuomotor perturbations (Contreras-Vidal and Buch, 2003; Messier et al., 2007; Paquet et al., 2008; Venkatakrishnan et al., 2011; Mongeon et al., 2013). Similarly, results from neuroimaging research has indicated that the contribution of the basal ganglia, and the striatum in particular, appears to be greatest during the initial adaptation stage (Seidler et al., 2006; Albouy et al., 2013c), and then progressively decreases as a function of training (Shadmehr and Holcomb, 1997; Krebs et al., 1998). One explanation for the increased activation during initial adaptation is that the striatum functions as an adaptive search mechanism that selects new sensorimotor representations that may be more appropriate for moving in the novel sensorimotor environment (Contreras-Vidal and Buch, 2003; Grosse-Wentrup and Contreras-Vidal, 2007; Scheidt et al., 2012). Successful selections are subsequently rewarded whereas unsuccessful selections are penalized, resulting in a reward-based learning algorithm thought to be implemented in the basal ganglia circuitry (Doya, 2000). In addition to increased striatal activation, the initial adaption also results in increased activation in frontal cortical areas, including the PFC (Shadmehr and Holcomb, 1997; Della-Maggiore and McIntosh, 2005; Anguera et al., 2007; Gentili et al., 2011). While the striatum may be involved in finding sensorimotor mappings suited for the novel, perturbed environment, the frontal cortex appears to inhibit previously learned or established sensorimotor mappings that are no longer appropriate (Shadmehr and Holcomb, 1999; Gentili et al., 2011).

## Aging and motor learning

### Motor sequence learning

#### Initial acquisition of motor sequences

During the fast learning phase of MSL paradigms, older adults demonstrate significant improvements in performance as a function of practice, suggesting that they can learn novel motor sequences (Howard and Howard, 1989, 1992; Daselaar et al., 2003; Shea et al., 2006; Spencer et al., 2007; Brown et al., 2009; Fraser et al., 2009; Rieckmann and Bäckman, 2009; Nemeth and Janacsek, 2010; Nemeth et al., 2010; Romano et al., 2010; Fogel et al., 2012; Wilson et al., 2012). However, under certain task conditions such as increased task complexity or explicit knowledge of the sequence, older adults, as compared to young adults, have demonstrated deficits in learning rate and magnitude (Curran, 1997; Howard and Howard, 2001; Feeney et al., 2002; Howard et al., 2004, 2008; Bennett et al., 2007, 2011; Rieckmann and Bäckman, 2009). For example, the complexity of the learned sequence can be increased when random movements are interleaved with the to-be-learned repeated elements (i.e., a movement sequence of *r4r1r3r2* where *r* represents a random element and the numbers represent components of the repeated finger sequence to be learned). Such an increase in sequence complexity has revealed a disproportionately negative influence on older adults (Curran, 1997; Feeney et al., 2002; Howard et al., 2004; Bennett et al., 2007). Similarly, providing explicit information about a repeating sequence, particularly when the sequence is long, appears to impede MSL in older adults, whereas it has a negligible or even facilitative influence on sequence learning in younger participants (Willingham and Goedert-Eschmann, 1999; Howard and Howard, 2001; Willingham et al., 2002).

It has been proposed that these deficits are the result of age-related decreases in cognitive functioning (Salthouse, 1996; Howard and Howard, 2001; Howard et al., 2004; Rieckmann and Bäckman, 2009). For example, performing the alternating serial reaction time (ASRT) task (i.e., *r4r1r3r2*) requires that non-adjacent elements of the sequence be linked as part of a repeating sequence. Decreases in cognitive processing speed will interfere with linking the non-adjacent elements, effectively hindering the learning process (Salthouse, 1996; Howard et al., 2004). Similarly, providing explicit information about a repeating sequence is thought to negatively influence learning because this information consumes additional cognitive/neural resources. The additional resources allocated to the explicit learning of the motor sequence may result in reaching the ceiling of cognitive processing capacity in older but not younger adults (Frensch and Miner, 1994; Howard and Howard, 2001; Rieckmann and Bäckman, 2009). In addition, Seidler and colleagues have reported a significant correlation between explicit sequence learning and working memory in both young and older adults (Bo et al., 2009). This suggests that age-related decreases in working memory contribute to the age-related deficits in the initial acquisition of motor sequences.

**Figure 1:**
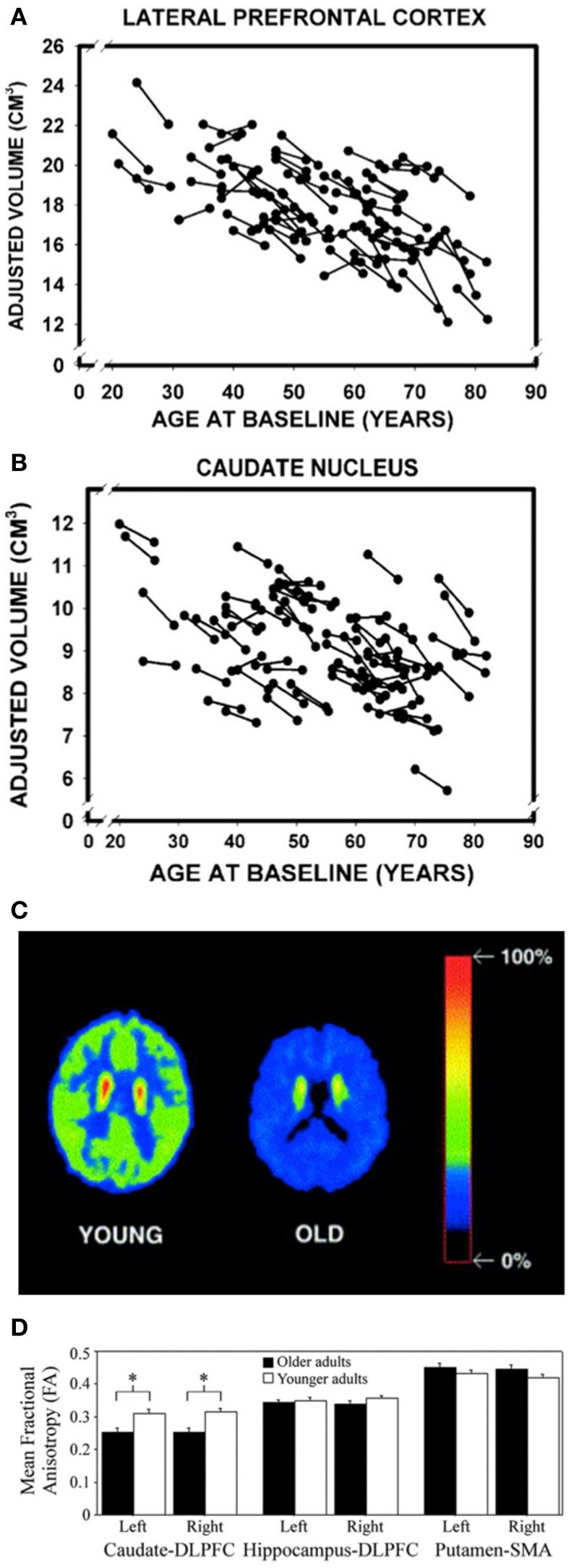
**Age-related volumetric declines in the (A) lateral prefrontal cortex and (B) caudate nucleus.** Reprinted from Raz et al. (2005), by permission of Oxford University Press. Similar volumetric decreases are also evident in the putamen (Raz et al., 2003). **(C)** Transaxial planes at the level of the caudate nucleus depicting decreased dopaminergic activity (relative uptake of a dopamine D2-like receptor ligand) in a representative older subject compared to a young participant. Reprinted from Neuroscience and Biobehavioral Reviews, 26, Kaasinen and Rinne (2002), with permission from Elsevier. **(D)** FA values for three different white matter tracts in younger and older adults. Reprinted from Neurobiology of Aging, 32, (Bennett et al., 2011), with permission from Elsevier. ^*^Significant age group differences (*p* < 0.01).

As both the frontal cortex and the striatum are heavily involved in the initial learning of motor sequences, these task-dependent behavioral deficits may be attributed to age-related degradations in cortico-striatal networks (Rieckmann and Bäckman, 2009; Rieckmann et al., 2010). Indeed, there are several pieces of evidence to support this explanation. First, substantial age-related structural changes are evident in both the frontal cortex and striatum, including reductions in volume (Figures 1A,B) (Gunning-Dixon et al., 1998; Raz et al., 2003, 2005; Hedden and Gabrieli, 2004; Allen et al., 2005; Kennedy and Raz, 2005). Second, aging is associated with significant decreases in dopamine (the prominent neurotransmitter acting in the basal ganglia), the presence of which has been shown to facilitate sequence learning and motor memory formation (Figure 1C) (Kaasinen and Rinne, 2002; Floel et al., 2005, 2008; Bäckman et al., 2006, 2010; Simon et al., 2011). Third, the integrity of the white matter tracts connecting the caudate nucleus and the dorsolateral PFC is decreased in older, as compared to younger, adults (Figure 1D) (Bennett et al., 2011). The caudate-DLPFC circuit is not only thought to be involved in forming associations between repeated elements that are necessary for early MSL (Jueptner et al., 1997; Poldrack et al., 2005), but degradations in this tract have also been related to age-related declines in sequence learning (Bennett et al., 2011). Fourth, implicit sequence learning in older adults is associated with decreased activation in the right putamen (Aizenstein et al., 2006). Interestingly, decreased activation in the putamen has also been observed in older adults during an interlimb coordination task (Van Impe et al., 2009) and proprioceptive stimulation (Goble et al., 2012), the latter of which was the result of age-related structural deficits. This decreased activation in the putamen is particularly surprising given that widespread age-related and task-dependent *increases* in activation are frequently reported (Mattay et al., 2002; Ward and Frackowiak, 2003). And, fifth, the pattern of brain activation during sequence learning in older adults suggests that the hippocampus may be compensating for disrupted striatal functioning (Rieckmann and Bäckman, 2009; Rieckmann et al., 2010). More specifically, in young adults, hippocampal activity decreases and striatal activity increases as a function of sequence learning (Schendan et al., 2003; Albouy et al., 2008), whereas in the older adults, activity in both the MTL, including the hippocampus, and the striatum *increases* (Rieckmann et al., 2010). The increased MTL activity may serve a compensatory function in order to maintain similar levels of performance despite age-related decreases in the structure and function of the striatum (Rieckmann and Bäckman, 2009; Rieckmann et al., 2010).

Collectively, these findings suggest that age-related degradations in the striatum contribute to the age-related deficits in the acquisition phase of MSL. When learning is implicit or when sequence complexity is relatively low, MSL is comparable to young adults due to compensation from other relevant neural structures, including the MTL, and the hippocampus in particular. However, in task conditions with an increased cognitive load (i.e., greater sequence complexity or explicit nature of the MSL task), the performance of older adults during the initial learning is *not* maintained, potentially due to an inability of the MTL and other neural substrates to compensate for age-related degradations in the striatum (Rieckmann and Bäckman, 2009).

It should be emphasized that degradations in the frontal cortico-striatal system are likely not the only neural correlate of impaired sequence learning in older adults. The initial phase of sequence learning is dependent on a widespread network of cortical and subcortical structures, including the frontal cortex, striatum, cerebellum and hippocampus. It is thus likely that age-related changes in these additional structures, particularly the hippocampus (e.g., Allen et al., 2005; Raz et al., 2005), contribute to the behavioral deficits. Interestingly, the pattern of brain activation during sequence learning in older adults (e.g., increased activation in *both* the hippocampus and striatum) (Rieckmann et al., 2010) was identical to that of a sub-group of participants in the experiment of Albouy et al. (2008) that demonstrated a decreased rate of sequence learning, suggesting that the interaction between the striatum and hippocampus may also contribute to sequence learning deficits in older adults.

#### Consolidation of motor sequences

Although older adults demonstrate significant savings in performance across multiple sessions, indicating retention of newly acquired motor memories for a period of up to 1 year (Shea et al., 2006; Spencer et al., 2007; Fraser et al., 2009; Nemeth et al., 2010; Romano et al., 2010; Wilson et al., 2012), the magnitude of the savings is less than that demonstrated by young subjects (Spencer et al., 2007; Brown et al., 2009; Nemeth and Janacsek, 2010; Nemeth et al., 2010; Wilson et al., 2012). Moreover, older adults fail to demonstrate the off-line gains in the absence of additional practice that are typically observed in young adults (Spencer et al., 2007; Wilson et al., 2012), suggesting that the consolidation process of motor memories following MSL is impaired in older adults.

Similar to the deficits in the initial acquisition of movement sequences, the deficits in MSL consolidation demonstrated by older adults can likely be attributed, at least partially, to age-related degradations in the striatum and/or hippocampus. In addition to the substantial age-related volumetric and dopaminergic declines discussed above (Gunning-Dixon et al., 1998; Kaasinen and Rinne, 2002; Raz et al., 2003, 2005; Hedden and Gabrieli, 2004; Floel et al., 2005, 2008; Kennedy and Raz, 2005; Bäckman et al., 2010), recent research in our lab investigating the role of sleep in MSL consolidation provides further evidence linking age-related changes in striatal activity to the motor memory consolidation deficits observed in older adults (Fogel et al., 2012).

In comparison to younger adults, older adults experience disrupted sleep, including increased sleep fragmentation and decreased sleep time and efficiency (Myers and Badia, 1995; Landolt and Borbely, 2001; Phillips and Ancoli-Israel, 2001; Huang et al., 2002). Despite spending more time in sleep stages 1 and 2, older adults have decreased amplitude, duration and number of sleep spindles (Landolt et al., 1996; Wei et al., 1999; Landolt and Borbely, 2001; Nicolas et al., 2001; Crowley et al., 2002). There is also growing evidence to suggest that spindles are involved in procedural memory consolidation (Fogel and Smith, 2006, 2011; Fogel et al., 2007; Nishida and Walker, 2007; Morin et al., 2008; Barakat et al., 2011, 2012). Moreover, a recent study has shown that in young subjects, activity in the putamen was increased following MSL and the increased activity was correlated with sleep spindles (Barakat et al., 2012). It is thus likely that impaired motor sequence consolidation demonstrated by older adults can be attributed to their disrupted sleep architecture as well as the interaction between sleep and the neural substrates subserving MSL consolidation (i.e., the corticostriatal system and hippocampus). In support of this hypothesis, a recent study in our lab examined the consolidation of an explicit motor sequence following a retention period containing either a 90-min daytime nap or equivalent period of wake. The aim was to investigate the associated changes in functional brain activity in young and older adults (Fogel et al., 2012) to better understand the neural correlates of the age-related deficit in MSL consolidation (Spencer et al., 2007; Brown et al., 2009). Results demonstrated that while young adults revealed enhanced behavioral performance following an afternoon nap, older adults did not. Moreover, spindles in the young group were related to increased changes in activation in the putamen from training to the post-nap retest. By contrast, sleep spindles in the older adults were related to increased activation in regions in the cortico-cerebellar loop, a neural network that, although involved in the initial acquisition of motor sequences, is not essential for motor sequence memory consolidation. Critically, these data provide a link between sleep spindles, the cortico-striatal system and enhanced consolidation in younger adults. No such beneficial relationship was evident in older adults, a finding that is likely the result of age-related degradations in both the cortico-striatal system and sleep architecture.

Similar to the discussion on the initial learning of motor sequences, the age-related declines in the hippocampus (Allen et al., 2005; Raz et al., 2005) may also contribute to deficits in sequence consolidation. Whereas “fast-learning” young adults demonstrate increased and decreased activation in the striatum and hippocampus, respectively, as a function of practice, both “slow-learning” young adults and older adults demonstrate increased activation in both substrates (Albouy et al., 2008; Rieckmann et al., 2010). These “slow-learning” young adults in the experiment of Albouy et al. (2008) also demonstrated impaired overnight consolidation, suggesting that the altered dynamics between the hippocampus and striatum may, at least partially, underlie the age-related deficits in motor sequence memory consolidation.

### Motor adaptation

#### Initial adaptation session in older adults

During exposure to various sensorimotor perturbations, older adults have demonstrated gradual reductions in movement errors, indicating that they can adapt to manipulations in the sensorimotor environment. However, results have consistently shown that the rate of adaptation and final level of performance are significantly worse in older adults, as compared to younger individuals (McNay and Willingham, 1998; Fernandez-Ruiz et al., 2000; Buch et al., 2003; Bock, 2005; Bock and Girgenrath, 2006; Seidler, 2006; Heuer and Hegele, 2008; Hegele and Heuer, 2010; Anguera et al., 2011). Despite the age-related differences *during exposure* to sensorimotor perturbations, older adults demonstrate equivalent or even larger aftereffects, as well as similar levels of transfer across behavioral tasks as compared to young adults, suggesting that aging does not result in impaired sensorimotor adaptation (Fernandez-Ruiz et al., 2000; Buch et al., 2003; Bock, 2005; Bock and Girgenrath, 2006; Seidler, 2007a; Heuer and Hegele, 2008; Hegele and Heuer, 2010).

The dissociation between the exposure and post-exposure phases appears paradoxical. But, it has been postulated that the performance during the post-exposure phase reflects the ability to adapt implicitly, or recalibrate, to novel changes in the environment, whereas performance during exposure to the perturbation reflects both implicit adaptation as well as the implementation of strategies utilized in response to the movement errors caused by the perturbation (Bock and Schneider, 2002; Buch et al., 2003). Within this context, implicit adaptation or sensory recalibration does not degrade with age. Conversely, the age-related differences evident in the exposure phase would result from deficits in cognitive, strategic control (McNay and Willingham, 1998; Fernandez-Ruiz et al., 2000; Bock and Schneider, 2002; Bock, 2005; Bock and Girgenrath, 2006; Heuer and Hegele, 2011; Heuer et al., 2011). Several pieces of evidence are used to provide support for this explanation. First, older, as compared to younger, adults fail to acquire equivalent explicit information about the nature of the sensorimotor perturbations, and this explicit information is correlated to performance during the exposure phase, but not to the magnitude of the aftereffects (Bock, 2005; Heuer and Hegele, 2008). This suggests that younger adults benefit from acquired explicit information during the exposure phase. Second, deficits during the exposure phase in older adults are related to degradations in measures of cognitive functioning, suggesting a role for cognitive processes during the exposure phase (Bock, 2005; Heuer and Hegele, 2008; Anguera et al., 2011; Langan and Seidler, 2011). For example, Seidler and colleagues have indicated that the inability to appropriately engage spatial working memory processes are correlated to the MA deficits observed in older adults (Anguera et al., 2011). This result is similar to their findings indicating that working memory deficits contribute to difficulties in the initial acquisition of movement sequences (Bo and Seidler, 2009; Bo et al., 2009). Third, when the potential use of explicit strategies is minimized by introducing the sensorimotor perturbation in gradual increments, age-related deficits during the exposure phase disappear (Buch et al., 2003; Cressman et al., 2010). These results thus suggest that age-related deficits in cognitive, strategic control, and not necessarily implicit MA, underlie the behavioral difficulties observed in older adults during exposure to sensorimotor perturbations.

The underlying neural substrates may help elucidate the dissociation described above between performance during the exposure and post-exposure phases. As discussed earlier, results from both patient and neuroimaging studies have implicated the cerebellum and striatum as key contributors to MA. Specifically, the cerebellum is thought to generate predictions of future states, and discrepancies between actual and predicted states are then used as error signals that drive the adaptation process by altering the synaptic weights between the PPC and M1 (Tseng et al., 2007; Tanaka et al., 2009). This cortico-cerebellar network would then be considered responsible for the implicit adaptation or sensory recalibration process that is reflected by the magnitude of the aftereffects when the perturbation is suddenly removed. The cortico-striatal network would operate in parallel, particularly during the early portions of the exposure phase when the magnitude of the movement errors is large (Venkatakrishnan et al., 2011; Mongeon et al., 2013). Specifically, the striatum is thought to function as an adaptive search mechanism that attempts to retrieve sensorimotor representations more appropriate for the perturbed environment (Contreras-Vidal and Buch, 2003; Grosse-Wentrup and Contreras-Vidal, 2007; Scheidt et al., 2012). The frontal cortex and the PFC in particular, would inhibit previously learned, established sensorimotor mappings that are no longer appropriate (Shadmehr and Holcomb, 1999; Gentili et al., 2011). This cortico-striatal network would then contribute, along with the cortico-cerebellar network, to the reduction of movement errors in the exposure phase. Within this context, the decreased performance observed during the exposure phase demonstrated by older adults would appear to be the result of impaired functioning of the cortico-striatal networks. In addition to the age-related decreases in dopamine and striatal volume that were highlighted in the section *Initial Acquisition of Motor Sequences* (Kaasinen and Rinne, 2002; Raz et al., 2003, 2005; Kennedy and Raz, 2005; Bäckman et al., 2006, 2010), the frontal cortex, and the PFC in particular, shrink substantially with age; and, there are robust degradations in the white matter tracts connecting the caudate nucleus and DLPFC (Allen et al., 2005; Hedden and Gabrieli, 2005; Kennedy and Raz, 2005; Raz et al., 2005; Bennett et al., 2011). Altogether, the present findings suggest that similar to the initial learning and consolidation of motor sequences, the age-related changes in the frontal cortico-striatal network likely contribute to the performance deficits evident in the exposure phase of MA paradigms.

As the magnitudes of the aftereffects are generally comparable in young and older adults, this would suggest that age-related degradations in the functioning of the cortico-cerebellar system are relatively minimal. However, the cerebellum does exhibit similar age-related declines as the striatum, at least with respect to reductions in volume (Luft et al., 1999; Raz et al., 2005). In addition, such degradations in the cortico-cerebellar system are thought to substantially contribute to age-related deficits in motor and cognitive functioning (e.g., Hogan, 2004). Thus, this raises the following question: why do older adults demonstrate comparable aftereffects despite substantial age-related declines in the cortico-cerebellar system? There are two potential, and certainly not mutually exclusive, possibilities. First, there is evidence to suggest that different types of MA depend on different regions of the cerebellum. Specifically, research on cerebellar patients suggests that the posterior lobe of the cerebellum is more involved in visuomotor adaptation, whereas the anterior lobe is more involved in force field paradigms (Rabe et al., 2009; Donchin et al., 2012). This finding is consistent with activation, as measured with PET, in the posterior lobe during visuomotor adaptation (Krakauer et al., 2004). There is also evidence suggesting that while there are significant age-related degradations in the cerebellum as a whole, the anterior lobe experiences substantial changes with age, including reductions in volume as well as granule and Purkinje cell numbers (Andersen et al., 2003). Age-related declines in the posterior lobe were less robust and tended to not reach significance. As the majority of MA research in older adults has employed visuomotor paradigms, the lack of substantial age-related deficits in the magnitude of the aftereffects is consistent with the notion that age-related degradations in the posterior lobe appear to be relatively minimal, effectively resulting in similar aftereffects in young and older adults. This explanation would then predict age-related differences in the magnitude of the aftereffects following force field adaptation, as this paradigm is more dependent on the anterior lobe of the cerebellum.

A second potential explanation is that as the majority of MA paradigms employ sensorimotor perturbations during the execution of goal-directed reaching movements, it could be argued that the adaptive processes underlying the traditional reach adaptation paradigm are relatively “simple” and are robust to the age-related degradations in cortico-cerebellar functioning. If task difficulty were increased, then age-related changes in the cortico-cerebellar system would result in more robust deficits at the behavioral level. Support for this hypothesis comes from a recent study in which older adults demonstrated reduced aftereffects in an adaptive locomotion task (e.g., split-belt paradigm) (Bruijn et al., 2012). The authors suggested that gait adaptation necessitates the reorganization of all body segments and that this increased task complexity, as compared to reaching adaptation paradigms, reveals age-related deficits in MA that are likely the result of degradations in the cortico-cerebellar networks (Bruijn et al., 2012). However, it should be emphasized that gait and posture are also more dependent on the anterior lobe of the cerebellum; thus, the age-related differences in Bruijn et al. (2012) may not be the result of task complexity *per se*, but may also be manifestations of the age-related degradations in the anterior cerebellar lobe noted above (Andersen et al., 2003). Regardless, the explanations presented above are speculative and additional research investigating the relationship between age-related degradations in cortico-cerebellar pathways and MA is necessary.

#### Motor adaptation retention

Surprisingly, retention following MA has not been as extensively examined in older adults. In a 5-year follow-up of the mirror-tracing task, older adults demonstrated savings in performance, although the magnitude of the savings was less than that of middle-aged and young adults (Rodrigue et al., 2005). However, older adults demonstrated significant transfer across different adaptation tasks (i.e., visual gain and rotation adaptation) and perturbation magnitudes when transfer was assessed 1–2 days after the initial training (Seidler, 2007a,b; Bock and Schneider, 2001). This facilitative effect was even more pronounced in the older subjects (Bock and Schneider, 2001). Collectively, this previous research potentially suggests that retention following MA is not impaired in older subjects. A lack of age-related behavioral deficits in MA retention, predominantly considered a function of the cortico-cerebellar network, would further suggest that the deficits observed in older adults during the exposure phase of MA paradigms are the result of age-related cortico-striatal, and not cortico-cerebellar, degradations. Again, however, a more in-depth investigation of this hypothesis is certainly necessary.

## Concluding remarks

The extant aging and motor learning literature has consistently reported that older adults have deficits in: (1) the initial acquisition of movement sequences under conditions of increased task complexity; (2) the consolidation of learned motor sequences; and, (3) the exposure, but not post-exposure, phase of MA paradigms. This review discussed evidence linking the behavioral deficits to age-related changes in relevant neural substrates. Specifically, the behavioral results are, at least partially, manifestations of age-related dysfunctions in the structure and functioning of the fronto-striatal networks subserving the different phases of the two motor learning paradigms.

An open question is what are the specific changes within the cortico-striatal network that result in the behavioral deficits discussed above? We have reviewed evidence indicating that the aging process is associated with decreased volume in the frontal cortex as well as the caudate and putamen (Raz et al., 2003, 2005; Allen et al., 2005), disruptions in the dopaminergic system (Kaasinen and Rinne, 2002; Bäckman et al., 2010, 2006) and degradations in the white matter tracts connecting the striatum to the frontal cortex (Bennett et al., 2011). Although these age-related neural changes have been associated with learning deficits in older adults (Kennedy and Raz, 2005; Paquet et al., 2008; Bennett et al., 2011), the specific influence of each of these neural changes on MSL and MA is not fully understood. Future research should attempt to disentangle the relative contributions of these age-related neural changes on motor learning, a task that is difficult as these changes occur in parallel.

Future research should also investigate conditions or interventions in which the potential for motor learning in older adults is facilitated. For example, given that the evidence reviewed here suggests that age-related changes in sleep may underlie the MSL consolidation deficits observed in the elderly, interventions to improve sleep quality in older adults may have a therapeutic benefit for motor learning. Research in young adults has also indicated that motor learning and consolidation is enhanced if participants avoid potentially interfering tasks immediately following training (Krakauer et al., 2005; Korman et al., 2007). This suggests that it may be possible to structure a training regimen that maximizes the probability of enhanced motor learning in older adults. A second potential avenue to enhance motor learning is non-invasive brain stimulation, such as transcranial direct current stimulation (tDCS). It has proven effective in facilitating motor learning, consolidation and retention in young adults across a range of tasks including MSL and MA (Galea et al., 2009, 2010; Reis et al., 2009; Nitsche et al., 2010). Last, action observation training has also contributed to motor memory formation (e.g., Stefan et al., 2005). Optimizing the potential for motor learning and experience-dependent brain plasticity in older adults will not only enhance the effectiveness of interventions aimed to mitigate age-related declines in motor performance, but can also be used to improve neurorehabilitative interventions for individuals with movement disorders or neurological injuries (e.g., Celnik and Cohen, 2004; Ertelt et al., 2007; Celnik et al., 2008, 2009).

In sum, we reviewed substantial evidence demonstrating degradations in neural structure and function associated with aging. It should be emphasized that these dysfunctions are not the result of passive processes that simply unfold as a function of age. Future research should continue to investigate potential experiences or therapeutic interventions, such as physical and mental activity regimens that may minimize the age-related neural degradations associated with the aging process (for review, see Seidler et al., 2010). Such investigations will promote the importance of specific experiences as an effective avenue to address a subset of the challenges introduced by our aging society.

### Conflict of interest statement

The authors declare that the research was conducted in the absence of any commercial or financial relationships that could be construed as a potential conflict of interest.

## Footnotes

^1^Our discussion of age-related behavioral deficits will be limited to motor *learning*. For a detailed discussion on age-related deficits in motor *performance*, please see Seidler et al. (2010).

^2^The notion of spontaneous, off-line enhancements has recently been a topic of debate. It has been suggested that off-line gains are manifestations of fatigue effects during the end of the initial training session (Brawn et al., 2010). However, recent results (Albouy et al., 2013a,b) demonstrated off-line gains even after controlling for fatigue.
